# DETERMINANTS OF AGE AT NATURAL MENOPAUSE AMONG POSTMENOPAUSAL GHANAIAN WOMEN IN OKAIKWEI NORTH DISTRICT, GHANA

**DOI:** 10.1093/geroni/igae098.3648

**Published:** 2024-12-31

**Authors:** Okyere Kontor, Veneranda Nyarko, Paul Botwe

**Affiliations:** University of Ghana School of Public Health, ACCRA, Greater Accra, Ghana; KORLE-BU TEACHING HOSPITAL, ACCRA, Greater Accra, Ghana; University of Ghana, Accra, Greater Accra, Ghana

## Abstract

The age at natural menopause, influenced by genetic and modifiable factors is linked to the risk of certain chronic conditions. While menopause has been studied extensively in other countries, there is limited data on its determinants among women in developing countries like Ghana, creating a theory gap for investigations. This study aims to assess post-menopausal women’s knowledge of menopause, determine the average age at natural menopause, and identify factors influencing this age among postmenopausal Ghanaian women in the Okaikwei North Municipality of Ghana. A cross-sectional study design with a quantitative approach was used. Consecutive sampling selected 296 post-menopausal women attending Achimota Hospital’s (municipal referral hospital) outpatient clinic. Data were collected via a structured questionnaire and analyzed using Stata version 16. Descriptive statistics, bivariate analysis (Chi-square and Fisher’s exact test) and multiple logistic regression (crude and adjusted odds ratios) were used, with statistical significance set at p < 0.05. The study found that 48.3% of participants had adequate knowledge about menopause. Most women experienced the “expected” natural menopause, 46-55 years (93.2%), with early menopause (40-45 years) at 3.7% and late menopause (>55 years) at 3.1%. The average age at natural menopause was 49.5 years (±0.17 SD). Significant factors associated with natural menopausal age included age at menarche, age at first pregnancy and contraceptive use. The study highlighting low knowledge about menopause among the participants therefore recommends an enhanced practical education, cost-effective health care, and women’s empowerment to better prepare women for menopause, particularly those approaching this transition.

